# Estrogenic activity in tampon products

**DOI:** 10.1210/jendso/bvag094

**Published:** 2026-04-17

**Authors:** Emma S Sutherland, Gihani Manodara, Ashley Gillon, Kelsey L Stevens, Alexia Kauff, Alison K Heather

**Affiliations:** Department of Physiology, School of Biomedical Science, University of Otago, Dunedin 9013, New Zealand; InsituGen Ltd., Dunedin 9013, New Zealand; Department of Physiology, School of Biomedical Science, University of Otago, Dunedin 9013, New Zealand; InsituGen Ltd., Dunedin 9013, New Zealand; InsituGen Ltd., Dunedin 9013, New Zealand; Department of Medicine, Otago Medical School, University of Otago, Dunedin 9013, New Zealand; Commercial Research & Development, ALS Food and Environmental NZ, Hamilton 3214, New Zealand; Department of Physiology, School of Biomedical Science, University of Otago, Dunedin 9013, New Zealand; InsituGen Ltd., Dunedin 9013, New Zealand; Department of Physiology, School of Biomedical Science, University of Otago, Dunedin 9013, New Zealand; InsituGen Ltd., Dunedin 9013, New Zealand

**Keywords:** estrogen bioactivity, endocrine-disrupting chemicals, tampons, cell-based bioassay

## Abstract

Tampons are widely used menstrual products with prolonged mucosal contact, raising questions about their potential role in exposing females to endocrine-disrupting chemicals (EDCs). This study evaluated estrogenic activity in tampon extracts using a cell-based estrogen receptor reporter bioassay. Synthetic and organic tampons sourced from domestic and international markets were tested for estrogenic bioactivity. Of the 18 brands analyzed, estrogenic activity was detected in nearly half, independent of material type. Comparative chemical analysis of one estrogenic-active brand vs one nonactive brand via high-resolution mass spectrometry tentatively identified various plasticizers, surfactants, and fragrance agents, that were present in higher concentrations in the estrogenic-active brand. The list of chemicals included known EDCs, such as phthalates and alkylphenols, that may be responsible for the observed estrogenic activity. Notably, estrogenic activity varied by brand, suggesting formulation-dependent risk. Although the detected in vitro activity levels were low, the findings demonstrate that compounds capable of activating estrogen receptors can leach from tampons. These results highlight the importance of including endocrine bioactivity assays in tampon safety assessments and suggest that safer formulations are achievable. Further investigation is warranted to assess long-term health implications of low-level EDC exposure from menstrual products.

Tampons are widely used menstrual products with reports of up to 85% of female individuals in Western countries favoring them over other products [1, 2]. In order to manage bleeding, tampons are inserted into the vagina for hours at a time resulting in prolonged contact with the highly vascularized vaginal mucosa. This contact promotes absorption through the walls of the mucosa bypassing hepatic first-pass metabolism. For certain compounds, such as estradiol, this leads to higher serum concentrations compared to oral administration [2, 3]. The high usage of tampons together with the intravaginal absorbency has led to recent questioning of the potential of tampons to act as a source of chemical exposure.

Tampons comprise a core absorbent material of cotton, rayon, or a blend of viscose/rayon/cotton. Tampons made of 100% cotton are marketed as organic products differentiating them from conventional tampons that are inherently more synthetic in composition. The core is contained by an outer layer that can be cotton veil, polyester, or polypropylene. The string used for vaginal removal is typically made of cotton, but in the synthetic brands can be a blend of polyester and polypropylene. The string is often bleached and coated with a surfactant to prevent fraying. During manufacture, functional agents can be added to the tampons, including plasticizers to make them more flexible, surfactants to assist in fiber processing and fluid absorption, fragrances and deodorants to provide scent, and lubricants and emollients to improve insertion comfort [4].

Across many years of menstruation, tampon use involves regular, repeated contact with the vaginal mucosa. A range of chemicals have previously been detected in tampons, including dioxins, furans, polycyclic aromatic hydrocarbons, phthalates, parabens, bisphenols, triclocarban, glyphosate, and volatile organic compounds [5, 6]. Metals(loids) have also been detected in tampons, including lead, arsenic, calcium, and zinc among others [7]. It is recognized that tampon functionality requires the inclusion of some chemical compounds to provide product integrity, absorbency, flexibility, and comfort [4]. Within this context, a core principle of sustainable chemistry is that such constituents should not include chemicals known to pose hazards. For products like tampons, even trace amounts of leaching during prolonged contact with the highly absorptive vaginal mucosa may be relevant. Therefore, the chemicals used in these products should be evaluated for endocrine-disrupting activity and other potential risks prior to their widespread use.

Endocrine-disrupting chemicals (EDCs) adversely impact on the normal functioning of the endocrine system. They can mimic, block or alter the signaling of natural hormones in the body, creating hormonal imbalance. Notably, EDCs can exert their effects at very low concentrations and may have significant effects on human physiology, especially during critical windows of development including fetal life, puberty, or pregnancy [8, 9]. Common examples of EDCs include certain plasticizers such as phthalates, industrial chemicals such as bisphenol A, pesticides like DDT, and personal care product ingredients such as parabens. Many EDCs exert estrogenic bioactivity that alters reproductive function, resulting in reduced ovarian reserve, endometriosis and uterine fibroids, and the disruption of menstrual cycles, as well as an increased risk of breast cancer [10-14].

Cell-based estrogen reporter assays, such as the T47D-luc estrogen bioassay (T47D-luc cells), are useful tools for detecting and quantifying estrogenic activity in complex chemical mixtures [15]. The T47D-luc cells have been genetically modified to express a luciferase reporter gene under the control of estrogen-responsive elements. When the naturally expressed estrogen receptors (ERs) bind estrogenic compounds, they activate the expression of luciferase by binding to the estrogen-responsive element and enhancing gene expression. The level of luciferase can be quantified as measurable luminescence, thereby providing a functional readout of ER activation. The key advantage of this approach is the ability to measure net biological effect of all estrogenic compounds present in a sample. This is especially valuable when assessing the complex mixture of plasticizers, surfactants, emollients, lubricants, and fragrance agents that may co-exist in tampon material. Many of these may only be weak agonists in isolation but potentially produce biological effects in combination [16, 17].

This study aimed to evaluate the estrogenic activity of synthetic and organic tampon extracts using the T47D-luc estrogen bioassay. We sought to determine whether differences in chemical content could explain differences in estrogenic bioactivity observed in vitro, and to explore what these findings might suggest about the potential for tampon-derived chemicals to interact with ERs.

## Materials and methods

### Tampon sample selection

Tampon products from multiple manufacturers, brands, product lines, absorbances, and countries of origin were selected for study. We tested a total of 18 unique brand-product-line-absorbency combinations, representing 13 brands, across synthetic and organic compositions (Table 1). All of the tampon brands were unscented (according to packaging labels or website information). We purchased tampons between July 2023 and October 2024 from supermarket stores in New Zealand, and from online retailers (for European and USA purchases). Tampons purchased in New Zealand were not the same products as purchased from the USA, although there was overlap for 2 brands (Table 1). One brand sent their products directly to the laboratory. We tested 2 tampons taken at random from each of the 18 products.

**Table 1:** Tampon brands tested in this study

Brand	Tampon material (as per label and/or website information)	Country of origin	Size	Estrogen activity detected
A	Certified organic cotton	New Zealand	Regular	No
B	100% non-chlorine bleached rayon polyester, polyethylene, polyester string	USA	Regular	No
C	100% certified organic cotton, including string. Wrapper made from cellulose	Europe	Regular	No
D	Rayon made from cellulose fibers, polyester, polyethylene, paraffin wax, pigment white 6 (Titanium dioxide)	USA	Regular	Yes
E	Top sheet 100% organic cotton, core FSC® certified material, plant-based	Australia	Regular	Yes
F	Chlorine-free bleached rayon (derived from plant materials), polypropylene/polyester cover, polyester/cotton string	Australia	Regular	Yes
G	100% certified organic cotton	Europe	Regular	Yes
H	100% certified organic cotton including veil, core, string. Paper wrapper, cardboard box	Europe	Regular	Yes
I	Organic cotton (core, string, water repellent wax)	Israel	Super	No
J	Organic cotton (core, string, water repellent wax)	Slovenia	Super	No
K	Organic cotton (core, string, water repellent wax)	Israel	Regular	No
L	Organic cotton (core, string, water repellent wax)	Israel	Super	No
M	Organic cotton	Europe	Regular	No
N	Rayon, cotton fiber, polyester, polsorbare 20, wax blend (paraffin, butyl stearate, carbauba wax, polymer wax dispersion)	USA	Regular	Yes
O	100% cotton core	USA	Regular	No
P	100% cotton core	USA	Regular	Yes
Q	Rayon, water, polyester, polyethylene, paraffin wax, pigment white 6 (titanium dioxide)	USA	Regular	Yes
R	Rayon, polyester, purified cotton	USA	Regular	No

### Sample preparation and analysis

Either 2 portions (1 g each) from the core tampon without string were cut from each tampon or one entire tampon (2.5 g) without string underwent testing. The tampons were soaked in 10 mL (1 g) or 25 mL (2.5 g) ethanol:milliQ water (50% v/v) for 4 hours then agitated by 10 minutes sonication, 40 minutes shaking (200-210 rpm), 10 minutes sonication, and then vortexing for 2 minutes. The extract was centrifuged for 15 minutes at 4000*g*. Five mL of the supernatant was then loaded into a prewashed SPE column (2 g Bond Elut, Agilent Technologies) that was preconditioned with 3 mL methanol, followed by 3 mL sodium acetate (pH 4.8). The column was washed sequentially with 2 mL milliQ water, 1.5 mL sodium carbonate (10% w/v), 2 mL milliQ water and 2 mL milliQ water/methanol (1:1 v/v). After washing, the column was air dried before the sample was eluted with 4 mL acetonitrile. The solvent was dried and sample extract reconstituted in 200 μL 100% ethanol (herein “samples” at 1 g/200 μL or 5 mg/μL). For bioassay testing, 2 μL of the sample was added to 198 μL DMEM F-12 phenol-red free medium. From this dilution, 100 μL was mixed with 100 μL DMEM F-12 phenol-red free medium. This dilution series was continued for 2 more dilutions and all 4 dilutions were tested (50 μg/μL, 25 μg/μL, 12.5 μg/μL, 6.25 μg/μL). For the blank control sample, 5 mL of 1:1 ethanol:water solution was loaded into the prewashed column and treated as per samples.

### Estrogen receptor bioassay

Estrogen Receptor Luciferase Reporter T47D Stable Cell Line cells (Signosis, Santa Clara, CA, USA: Cat # SL-0002, T47D-luc)) were grown to 90% confluence in a 550 mL cell culture flask with RPMI 164 medium supplemented with 10% (v/v) Hyclone fetal bovine serum (GE Lifesciences) and Geneticin (75 μg mL^−1^, G148, Life Technologies). Cells were then seeded at 5 × 10^4^ cells per 100 μL DMEM F-12 phenol-red free medium per well of a 96-well culture plate. The medium was supplemented with charcoal-stripped fetal bovine serum (FBS) (10% v/v) and G148 (75 μg mL^−1^). Cells were then incubated at 37 °C for 24 hours under 5% CO_2_.

For estradiol standards preparation, a 2μM stock solution of estradiol (Sigma-Aldrich, E8875) in 100% ethanol (same as extracts) was diluted as 15 μL in 285 μL of DMEM F-12 phenol-red free medium. From this 100nM stock, 100 μL was added to 200 μL DMEM F-12 phenol-red free medium. This 1:3 dilution was continued for 10 more dilutions, and all 11 dilutions were used to generate the estradiol dose-response curve.

For treatment with estradiol standards or samples as diluted above, medium was removed from the cells by aspiration and replaced with 90 μL of fresh DMEM F-12 phenol-red free medium, supplemented with charcoal-stripped FBS (2% v/v) and G148 (75 μg/mL). Then 10 μL of the estradiol standards or the sample dilutions were added to 90 μL medium and the cells were incubated at 37 °C for 24 hours under 5% CO_2_. Final sample concentration represented (1) 5 μg/μL, (2) 2.5 μg/μL, (3) 1.25 μg/μL, and (4) 0.625 μg/μL or a 1:1000 dilution of the original extract.

After incubation, media was aspirated from all wells and 100 μL BrightGlo substrate (Promega) was added. After a 2-minute incubation at room temperature, the BrightGlo was transferred to a white opaque 96-well plate and luminescence reported using a SpectraMax i3x plate reader (Molecular Devices).

### Liquid chromatography–high-resolution mass spectrometry protocol

Extraction of tampons was performed as above. For analysis, the 200 μL extract from 7 tampons of Brand A or D were combined in order to have the extract volume needed for HRMS analysis. Ethanolic extracts were transferred to an HPLC vial and diluted with a 1:1 liquid chromatography–mass spectrometry compatible solvent (0.1% formic acid) and 10 μL injected into the liquid chromatography–high-resolution mass spectrometer (LC-HRMS). A sample of ethanol was treated as above and used as a blank. Separation was performed with an Ultimate 3000 HPLC coupled to a Thermo Exploris 480 Orbitrap Mass Spectrometer. Liquid chromatography separation was performed on an Acquity UPLC BEH C18 column (100 × 2.1 mm ID, 1.7 μm, Waters) at 55 °C in a linear gradient (20%-95% over 29 minutes) with water and methanol as the mobile phases, both containing 0.1% formic acid. The mass spectrometer was equipped with an electron spray ionization source (H-ESI) operated in positive and negative mode with parameters described in Table 2. Data were acquired over the mass range of 100 to 1000 Da using full scan MS mode at 120 000 resolution in conjunction with an internal mass calibration (EASY-IC). This allowed a nontargeted approach to the analysis. In addition, data dependent mass scan (ddMS2) was used to collect fragmentation data at 15 000 resolution at 30 and 80 V HCD collision energy. Sensitivity of known standards, likely to be observed in sample (Table 3), were checked to ensure optimal ionization and separation parameters.

**Table 2:** Orbitrap MS parameters

Parameter	Setting
Positive ion (V)	3400
Negative ion (V)	2000
Sheath gas (Arb)	5
Sweep gas (Arb)	5
ITT (°C)	350
Vaporizer (°C)	400
AGC target	Standard

Abbreviations: ITT, intention to treat; MS, mass spectrometry.

**Table 3:** EDC reference analytes in 100 ppb standard used for Level A identification

Dimethyl phthalate (DMP)	Methyl Paraben
Diethyl phthalate (DEP)	Ethyl Paraben
Butyl benzyl phthalate (BuBzP)	Propyl Paraben
Di-n-pentyl phthalate (DNPP)	Levonorgestrel
Di-n-hexyl phthalate (DnHxP)	Ethinyl Estradiol
Di-n-heptyl phthalate (DnHpP)	Estriol
Di(2-ethylhexyl) phthalate (DEHP)	Estrone
Di(n-octyl) phthalate (DNOP)	Estradiol
Diisononyl phthalate (DINP)	Bisphenol A
Diisodecyl phthalate (DIDP)	
Bis(2-ethylhexyl) adipate (DEHA)	

Abbreviations: EDC, endocrine-disrupting chemical; ppb, parts per billion.

Data generated from the LC-HRMS were processed using Compound Discoverer 3.3 SP2 application (Thermo Scientific). Retention times were automatically aligned, and peak assignment was performed with a mass tolerance of 5 ppm and retention time tolerance of 0.1 minute. The workflow searched for multiple adducts as well as isotope pattern detection (Br;Cl). Identification was done by a local database and other data sources such as MassList, Chemspider, and mzCloud.

### Analysis

Estrogen receptor bioassay luminescence values were first corrected by subtracting the mean signal from the blank control wells across all dilutions. Estradiol was used as a positive control to generate a standard curve using a 4-parameter logistic regression to fit the data (0.001nM-100nM) (PRISM Graphpad v10). For samples, each tampon was tested as duplicate 1-g subsamples. Dependent on the experiment, 2 or 3 tampons from each brand were tested in total across 2 or 3 independent assays performed on different days. Each subsample was assayed in duplicate for each of the 4 dilutions tested. Subsamples were averaged to provide the mean per tampon. Statistical significance between treatments was assessed using one-way ANOVA followed by Tukey's post hoc test, with *P* values of < .05 considered significant. The limit of detection was defined as the mean of the vehicle control plus 3 times the SD. Samples eliciting responses above the limit of detection were classified estrogenically active. All analyses were performed using Graphpad PRISM v10.4.1.

## Results

### Characterization of the T47D-luc estrogen bioassay

**Figure 1:**
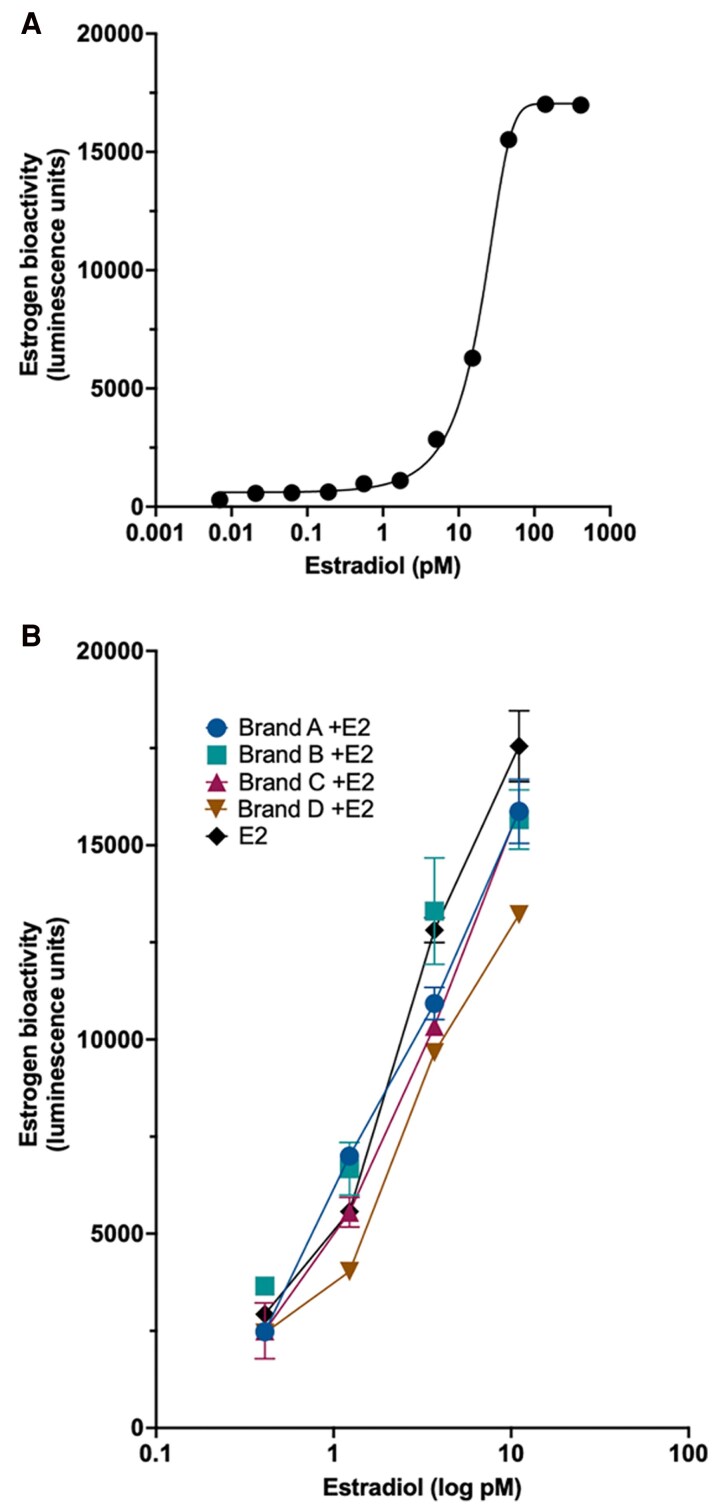
Estradiol dose-response and recovery of spiked estradiol from tampon extracts. (A) T47D-luc cells were treated with an 11-point estradiol dilution series starting at 30nM, followed by 10nM, 3.3nM, 1.11nM, 370pM, 123pM, 41pM, 14pM, 4.6pM, 1.5pM, and 0.5pM). For each concentration, 2 μL of steroid dilution was added to 198 μL assay medium, and cells were incubated for 24 hours. Estrogen-responsive luciferase activity was quantified using BrightGlo substrate. Luminescence values represent the mean of 4 technical replicates, and a sigmoidal dose-response curve with EC_50_ determination was generated using GraphPad v10. (B) Recovery of estradiol from tampon brands A-D following spiking and extraction. One gram of each tampon from Brands A, B, C, and D were spiked with 2nM estradiol, soaked in a 1:1 water:ethanol solution for 4 hours, extracted, evaporated to dryness, and reconstituted in methanol. Extracts were diluted 1:100 in cell culture medium and then serially diluted 1:2 to generate 4 assay points. T47D-luc cells were incubated with a further 1:10 dilution of each tampon extract for 24 hours and luciferase activity measured using BrightGlo. Each point represents mean ± SEM of 4 technical replicates corrected for the blank control sample (1:1 ethanol:water solution SPE-extracted as per samples). The data for the spiked tampons are plotted alongside estradiol standards run in parallel.

The sensitivity and dynamic range of the T47D-luc cells were evaluated to determine the suitability for detecting estrogenic activity in methanolic tampon extracts. Figure 1A shows a clear sigmoidal dose-response curve for estradiol (E2), spanning sub-picomolar to nanomolar concentrations. The calculated EC_50_ for T47D-luc cells under our experimental conditions was approximately 14.5pM. The bioassay demonstrated a limit of detection at approximately 0.5pM in keeping with a previous report [18].

### Estrogenic activity measured in E2-spiked tampons

In order to demonstrate that the T47D-luc cells could detect estrogenic activity from tampon extracts, 4 tampons from 4 different brands were spiked with estradiol at a concentration of 2nM. Each tampon was then soaked in a 1:1 water:ethanol solution for 4 hours, mimicking the recommended duration of tampon use in females. After soaking, the spiked tampons underwent the sonication, extraction, and dilution protocol before being tested for estrogenic activity using the T47D-luc assay.

Figure 1B illustrates the estrogenic activity observed from 4 tampon brands spiked with estradiol. The results demonstrate that the assay is able to accurately detect estrogenic activity from the tampon exudates after extraction and dilution (11.1, 3.7, 1.23, 0.41 picomolar). Detected estrogenic activity levels closely resembled those of estradiol alone. Specifically, at the highest dilution tested, estrogen recapture efficiencies were determined to be 93.5% for Brand A, 92.8% for Brand B, 94.3% for Brand C, and 80.5% for Brand D. These values confirm estradiol recovery across brands, with only modest attenuation in Brand D. Together, the data show that the estrogen bioassay accurately detects low-picomolar estrogenic activity even after tampon matrix extraction and processing.

### Estrogenic activity measured in tampon extracts sourced from New Zealand supermarkets

**Figure 2:**
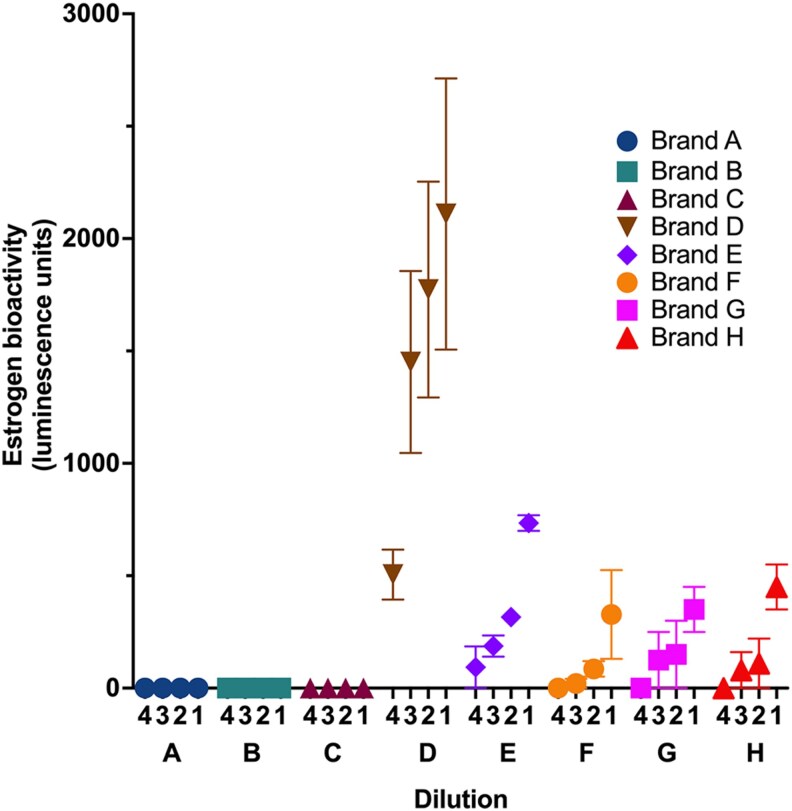
Estrogenic activity detected in extracts from 8 commercially available tampon brands (A-H). T47D-luc cells were exposed to methanolic extracts prepared from 1 g of core tampon material (string removed) from 8 tampon brands sold in New Zealand. Each 1 g sample was soaked in a 1:1 ethanol:water solution for 4 hours, sonicated, filtered, extracted, and then evaporated to dryness, and reconstituted in methanol. The final extract was diluted into assay medium at 1:1000 (1), followed by serial dilutions to generate 1:2000 (2), 1:4000 (3), and 1:8000 (4) exposure concentrations. Cells were incubated with each dilution for 24 hours, and estrogen-responsive luciferase activity was quantified using a luminescence plate reader. Data are presented as the mean ± SEM of 2 independently extracted tampons per brand, each tested as 2 portions of 1 g each, and each dilution tested with 4 technical replicates.

We next evaluated estrogenic activity from tampon extracts across 8 brands sourced from supermarkets in New Zealand, except for Brand A, which was provided directly by the manufacturer (Table 1). Two tampons per tampon packet were tested, with each tampon tested in duplicate as 2 portions (1 g each) cut from the same tampon. The results presented in Fig. 2 indicate that extracts from Brands A, B, and C exhibited no detectable estrogenic activity, indicating that both tampons tested from these brands did not leach measurable estrogen-mimicking compounds under the experimental conditions. In contrast, Brands D through H demonstrated clear estrogenic bioactivity, the levels of which varied across brands and predictably decreased with increasing dilution (dilution 1 [5 μg/μL] representing the highest concentration and dilution 4 [0.625 μg/μL] the most dilute). Although some variability is observed at the lowest end of the assay detection, this does not detract from the overall finding: the same brands consistently generated measurable estrogenic activity across independent tampons, extraction replicates, and assay days. The variation reflects operation near the assay's lower dynamic range, not inconsistency in the underlying biological signal.

### Testing batches of 2 selected tampon brands for estrogenic activity

**Figure 3:**
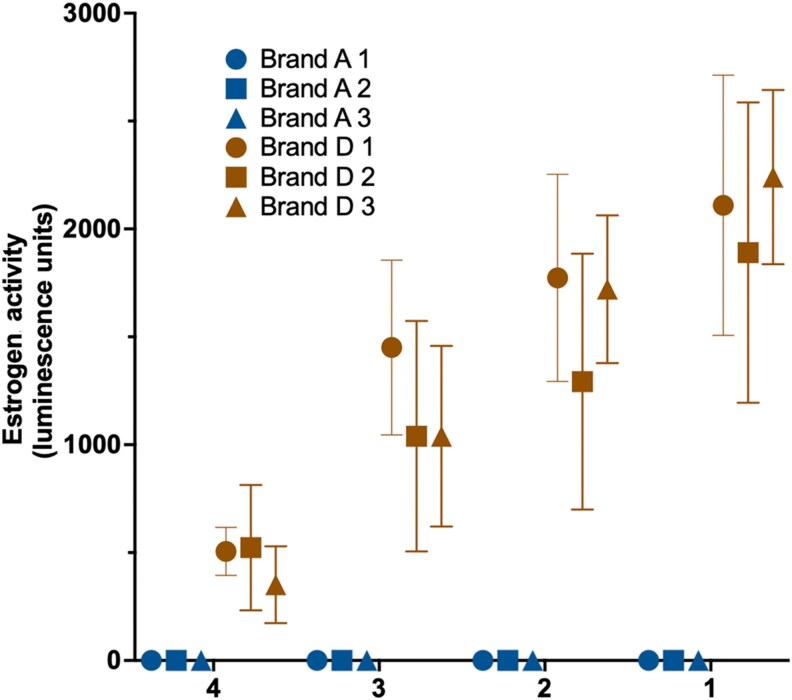
Independent batches of Tampon Brand D show estrogenic activity. T47D-luc cells were treated with methanolic extracts generated from 1 g of core tampon material (string removed) from 3 independent batches of Brand A and Brand D. For each batch, 3 separate tampons were extracted independently. Each 1 g sample was soaked in 1:1 ethanol:water solution for 4 hours, sonicated, filtered, extracted, and evaporated to dryness, and reconstituted in methanol. Reconstituted extracts were diluted into assay medium at 1:1000 (1), followed by serial 1:2 dilutions to produce 1:2000 (2), 1:4000 (3), and 1:8000 (4) exposure concentrations. Cells were incubated for 24 hours, and estrogen-responsive luciferase activity was quantified by using a luminescence plate reader. Data are shown as mean ± SEM of 3 independently extracted tampons per batch, tested as 2 portions of 1 g each with each dilution tested as 4 technical replicates.

We next analyzed 3 separate batches of 2 selected brands: one that had been determined to lack estrogenic activity (Brand A) and one positive for estrogenic activity (Brand D). Three tampons from each batch were tested on independent days, with each tampon tested as 2 1-g portions. For Brand A, no estrogenic activity was detected across all 3 batches, supporting the conclusion that Brand A does not contain detectable levels of estrogenic compounds under the experimental conditions tested (Fig. 3). Conversely, all 3 batches of Brand D exhibited estrogenic activity, albeit with some intra-batch variability.

### Estrogenic activity in internationally sourced tampon brands

**Figure 4:**
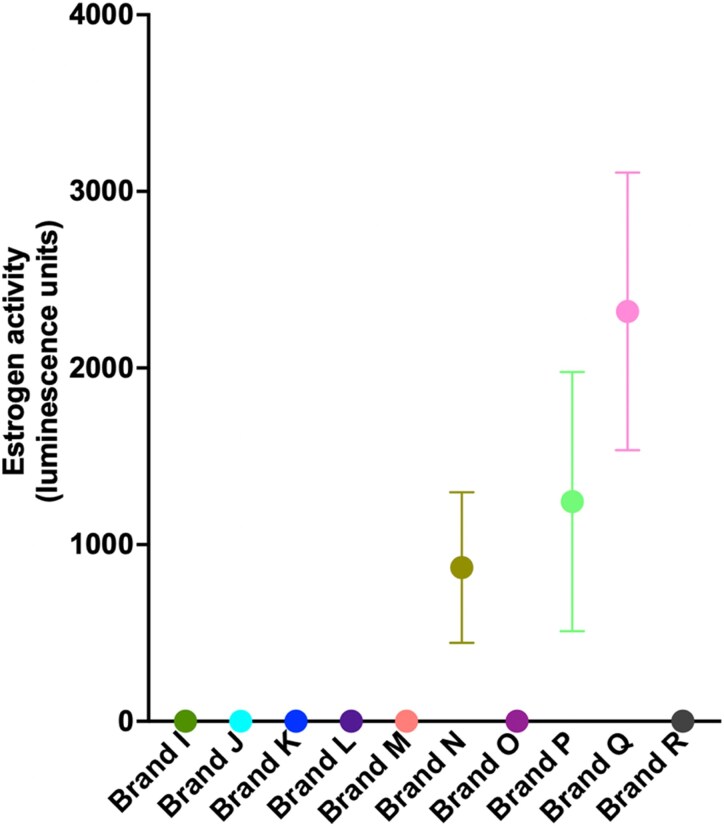
Estrogenic activity of internationally sourced tampon brands. T47D-luc cells were treated with methanolic extracts prepared from intact tampons (string removed) from a panel of internationally sourced tampon brands (Brands I-R). For each brand, 2 or 3 independent tampons were extracted separately on independent days. Each tampon was soaked in a 1:1 ethanol:water solution for 4 hours, sonicated, filtered, extracted, evaporated to dryness, and reconstituted in methanol. Reconstituted extracts were diluted into assay medium at 1:1000, and cells were incubated for 24 hours before estrogen-responsive luciferase activity was quantified using a luminescence plate reader. Data are shown as mean ± SEM of 2 or 3 independently extracted tampons per brand with each dilution tested as 4 technical replicates.

The data obtained thus far were from tampons sourced from New Zealand supermarkets. We next tested brands that were purchased internationally. For each brand, 2 to 3 tampons were tested (2 for negative tampons, 3 for any tampon brand that tested positive), with intact tampons tested. Figure 4 shows that Brands I to M, O, and R showed no measurable estrogenic activity. Brands N, P, and Q showed measurable estrogenic activity.

### Estrogenic activity in tampon brands with and without sonication

To help release any potential estrogenic chemicals into the tampon leachate, our processing of the tampons involved sonication after soaking for 4 hours. Sonication itself did not cause destruction of the tampon with the core remaining intact; however, it is a step that does not reflect the real-time use of tampons. To determine if sonication artificially created an estrogenic profile for the tampon extracts that would not physiologically occur, we compared the estrogenic activity of Brand A and Brand D after processing with and without a sonication step.

**Figure 5:**
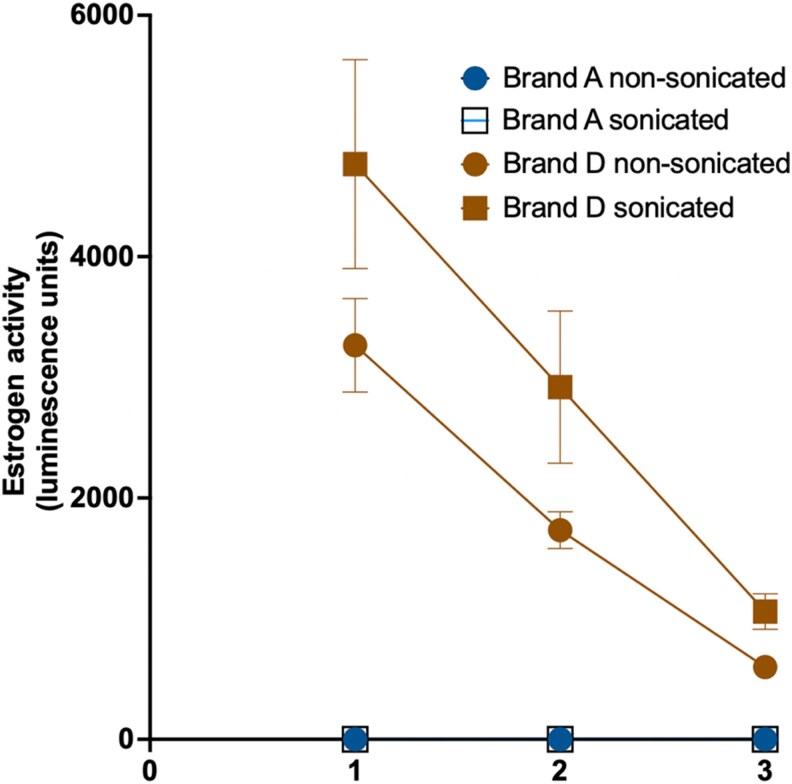
Effect of sonication on estrogenic activity of tampon extracts. T47D-luc cells were exposed to methanolic extracts prepared from 2 independent tampons per brand (Brands A and D, string removed), comparing extraction with and without sonication. Each tampon was soaked in a 1:1 ethanol:water solution for 4 hours, either sonicated or left nonsonicated, then filtered, extracted, evaporated to dryness, and reconstituted in methanol. Extracts were diluted into assay medium at 1:1000 (1), followed by serial 1:2 dilutions to generate 1:2000 (2) and 1:4000 (3) exposure concentrations. Cells were incubated for 24 hours, and estrogen-responsive luciferase activity was quantified. Data represent mean ± SEM of t2wo independently extracted tampons per condition with each dilution tested as 4 technical replicates.

Brand A (3 intact tampons tested across 3 individual testing days) showed no estrogenic activity either with active or absent sonication treatment (Fig. 5). Brand D (tested as for Brand A) exhibited estrogenic activity with no sonication, although this process did increase estrogenic activity, suggesting that agitation does promote greater chemical leaching (Fig. 5). Together, the results show that passive leaching occurs with forced agitation simply accelerating the process.

### Tentative identification of estrogenic chemicals in tampons exhibiting positive estrogenic activity

Chemical analyses of tampon extract injections (samples A and D) were compared to the control blank (ethanol) using LC-HRMS. Thousands of unique signals or peaks were detected within the wide mass range, but only well-defined peaks (rating ≥6.5) and those not present in blanks, were retained. Tentative identities for the remaining features were proposed where there were good matches to exact mass and isotope distribution patterns for known compounds in databases. The most abundant species were confirmed by matching MS2 fragmentation patterns to various databases. Only features with strong database matches (FISh score >40) were considered to be reputable peaks.

Statistical analysis enabled comparison between groups for each identified peak and between group differences (log2changes) over a threshold of 5 were evaluated, as these are compounds that were likely to be enriched in one sample. Peak areas and tentative identities are listed in Table 4, providing a comparative view between samples, although direct comparison between different compounds is limited due to variability in ionization efficiency. Levels have been assigned to each tentative identification based on the confidence demonstrated by the software output and chemical understanding. Level A indicates a high probability of correct identification due to correlation to a concurrently prepared known standard (Table 3), B indicates a match in multiple data sources and a FISh score >40, C indicates multiple matches with a less confident alignment of data, and D is an unidentified peak with significant area contribution. Confirmation of all species in levels B-D would require further fragmentation pattern analysis and retention time comparison with a known standard [19]. Table 4 shows that Brand D exhibited a heightened number of putatively identified species, showing substantially higher peak areas than for Brand A.

**Table 4:** Chemicals tentatively identified in tampon extracts by peak area: Brand A vs Brand D

Tentative identification	Function/Use	EDC activity	Level	Formula	Brand A	Brand D
Di-n-butyl phthalate	Plasticizer	Known EDC [20, 21]	A	C16 H22 O4	2.7E8	3.7E8
Benzyl butyl phthalate	Plasticizer, Solvent & Fixative	Known EDC [22]	A	C19 H20 O4	4.6E7	5.1E7
Phthalic anhydride	Plasticizer	Potential EDC [21]	B	C8 H4 O3	7.9E8	1.1E9
Tributyl citrate acetate	Plasticizer, phthalate alternative	Anti-Estrogenic [23]	B	C20 H34 O8	3.0E8	3.1E9
Diethyleneglycol dibenzoate	Plasticizer & viscosity modifier	Potential EDC [24]	B	C18 H18 O5	1.5E7	5.3E8
Dipropyleneglycol dibenzoate	Plasticizer & viscosity modifier	Known EDC [24]	B	C20 H22 O5	<1.0E7	5.1E8
N-Methyl hexadecanamide	Lubricant/emollient	Weak EDC [25]	B	C17 H35 N O	<1.0E7	5.5E9
Bis(2-ethylhexyl) sebacate	Plastizer and lubricant	Unknown	B	C26 H50 O4	1.9E7	1.1E8
Dibutyl succinate	Plasticizer and solvent	Known EDC [26]	B	C12 H22 O4	<1.0E7	4.8E7
Methyl cinnamate	Fragrance agent	Potential EDC [27]	B	C10 H10 O2	7.4E7	2.3e10
Cinnamic Acid	Fragrance agent	Unknown	B	C9 H8 O2	1.2E8	4.9E9
Pentadecane sulfonic acid	Surfactant & detergent agent	None	B	C15 H32 O3 S	<1.0E7	8.5E8
1-tetradecanol sulfonic acid	Surfactant, Emollient	Unknown	B	C14 H30 O3 S	<1.0E7	3.9E8
2-(Octylphenoxy)ethanol	Surfactant, emulsifier	Unknown	B	C16 H26 O2	1.2E7	3.9E8
Ethyl palmitoleate	Flavoring	Unknown	C	C18 H34 O	<1.0E7	2.0E9
Dioctyl adipate	Plasticizer	None	C	C22 H42 O4	6.2E7	3.4E8
12-Hydroxystearic acid	Stabilizer and emulsifying properties	None	C	C18 H36 O3	1.1E7	5.7E8
Myristyl lactate	Emollient and skin conditioning agent	None	C	C17 H34 O3	<1.0E7	3.4E8
Linoleyl alcohol	Emollient, lubricant	Unknown	C	C18 H34 O	<1.0E7	4.9E8
Diethylhexyl succinate	Emollient & Texture enhancer	None	C	C20 H38 O4	<1.0E7	2.1E8
Unidentified			D	C21 H28 O3	3.3E8	1.5E9
Unidentified			D	C16 H31 F3 O5	4.4E6	1.6E9
Unidentified			D	C11 H23 F N2 O3	1.8E8	3.6 E6
Unidentified			D	C13 H22 O5	9.4E5	3.8E8

Abbreviation: EDC, endocrine-disrupting chemical.

Table showing tentative chemical identity and their peak area detected in tampon extracts. Levels have been assigned to each tentative identification (A-D) based on the confidence demonstrated by the software output and chemical understanding. Level A indicates a high probability of correct identification due to correlation to a standard retention time, B indicates a match in multiple data sources and a FISH score >40, C indicates multiple matches with a less confident alignment of data, D indicates an unidentified peak with significant area contribution. Confirmation of all species would require further fragmentation pattern analysis and retention time comparison with a known standard [19].

## Discussion

This study has demonstrated that 8 of 18 tampon brands harbor estrogenic compounds that leach out of the material when soaked over a 4-hour period. The positive tampons included both synthetic and organic brands. Detailed mass spectrometry analysis suggests that one positive tampon brand had more known or suspected estrogenic EDCs, including phthalates, 4-tert-octylphenol (OP), 2-octophenoxy ethanol (OPE), hydroxy-1,4-benzoquinone, dioctyl adipate (DEHA) and other plasticizers, fragrances, and surfactant derivatives. These EDCs may contribute to the estrogenic bioactivity reported for this brand. We also note that all 3 batches of a positive brand assayed exhibited estrogenic activity. Together, the findings show that unwarranted estrogenic bioactivity can be detected from the leachate of tampons in a brand-specific manner.

The extracts from 18 tampons were tested for estrogenic bioactivity using the well-characterized T47D-luc cell-based bioassay [18]. This cell line represents breast cancer cells that are exquisitely sensitive to estrogenic compounds. The cells were used to test for estrogenic activity in extracted tampon leachate, after they had been sonicated and soaked in an ethanolic solution for 4 hours. To ensure the tampon processing steps were suitable for extracting estrogenic compounds, we tested estradiol-spiked tampons and showed for 3 of four brands that subsequent estrogenic bioactivity was >92% of that expected for the spiked concentration of E2. For the fourth brand, Brand D, E2 recapture efficiency was 80.5%; however, it was later shown that this tampon brand harbored estrogenic EDCs and therefore the decrease in E2-induced activity was likely competitively inhibited by leached estrogenic compounds or antagonized by the presence of anti-estrogens leached into the extract.

The nature of cell-based estrogenic bioassays is that they measure net effect of all the estrogenic compounds that are present in a sample. This allows the measurement of estrogenic activity from a complex mixture, including additive and synergistic effects. Comparing the percentage of ER bioactivity of tampon extract to the E2 curve, and allowing for the 25-fold concentration of the tampon extract, the E2 equivalence for the average measured estrogenic activity from the tampons is speculatively 62.5pM (17 ng/L). This most likely represents the summed estrogenic activity from several chemicals, rather than the single effect of just one chemical [16]. Individually, these chemicals may not exert strong estrogenic effects, but collectively reach the threshold needed to activate ER-mediated pathways [16]. This likely explains why Brand A was negative for estrogenic bioactivity despite the likely presence of a small number of potential EDCs in the HRMS analysis.

The estrogenic bioactivity measured in the bioassay may be explained by the presence of several known and suspected estrogenic EDCs as putatively identified for Brand D. Phthalates and various plastic additives, surfactants, fragrance and scent-masking agents, as well as lubricant/emollients, were all tentatively identified using HRMS. The presence of phthalates in tampons is in keeping with a previous study that specifically targeted phthalate detection [28]. Phthalates are known for their weak estrogenic effects and strong anti-androgenic effects. Common phthalates, including butyl benzyl phthalate (BBP) and di-n-butyl phthalate (DBP) detected in this study, induced breast cell proliferation, a hallmark of ER agonism at high concentrations [29]. The same chemicals can also interfere with aromatase and other steroidogenic enzymes in vitro, potentially altering estrogen synthesis and therefore affecting blood E2 levels [30]. These in vitro effects are echoed in human studies, where phthalate exposure has been linked to hypoestrogenism, diminished ovarian reserve, increased risk of premature ovarian failure [31], and uterine fibrosis [32-34]. With the growing awareness of health risks associated with phthalates, manufacturers have been switching to plasticizers with potentially more favorable profiles. Alternative plasticizers suggested by HRMS analysis include diethyl succinate, isophorane, tributyl citrate acetate, dibutyl sebacate, diethylene glycol dibenzoate, dipropylene glycol dibenzoate, tributyl phosphate (TBP), dioctyl adipate (DEHA), bis(2-ethylhexyl) sebacate (DEHS), dimethyl sebacate, and dibutyl succinate. Of these, DEHA at high concentrations was reported to increase breast cell proliferation, in similar fashion to phthalates [35], and DEHS and TBP are both characterized as anti-androgenic, suggesting they may be weakly estrogenic [36]. Cinnamate compounds also have weak estrogenic bioactivity due to their structural similarity to endogenous estrogens [27]. Octylphenol derivatives were also present in the tampon extracts and these are known to exhibit strong estrogenic activity, even at low concentrations [37]. In vivo, octyphenol exposure induces vaginal mucification and histological changes in ovarectomized rats [38].

We provide here the first evidence that compounds leaching from some brands of tampons have demonstrable estrogenic activity using a human ESR1/2-expressing breast cancer cell line. The estimated estrogen equivalence of 62.5pM or 17 ng/L leached from 1 g of each tampon is within the range of concern for chronic exposure. Such concentrations can trigger low-dose, non-monotonic effects, as has been reported for a number of estrogenic EDCs [17, 36, 39]. Some of the more studied EDCs have been shown to have the potential to bioaccumulate in tissues, raising the possibility of cumulative exposure over time [40, 41]. However, at present, the impact of estrogenic EDC-leaching tampons on human physiology or pathophysiology is not known.

An encouraging finding from this study is that tampons can be manufactured free of detectable estrogenic activity. The lack of estrogenic activity in extracts from some organic and synthetic tampons supports a brand-dependent chemical exposure model. While tampon manufacture requires the use of processing chemicals, finishing agents, and packaging that all can harbor estrogenic EDCs, several brands showed no measurable estrogenic activity. This demonstrates that formulations aligned with sustainable chemistry principles are achievable. By simplifying chemical profiles and minimizing concentrations of potentially hazardous compounds, some brands have already demonstrated that products can be successfully brought to market with no detectable estrogenic activity as measured by this assay. The findings raise important questions about regulatory oversight of menstrual products, particularly regarding the use of plasticizers, surfactants, and fragrance ingredients known or suspected to be hormonally active. There is a need for labeling requirements for such components in tampons, and the potential need for product safety testing that considers endocrine endpoints, not just irritation and infection risk. The clear difference in estrogenic bioactivity between the different brands reported here suggests that safer formulation is achievable.

In summary, estrogenic activity was demonstrated in 8 tampon extracts, representing 44% of those tested, in a brand-specific manner. As multiple known and unidentified estrogenic compounds likely exist in tampons, it will be important to use estrogen receptor bioassays of the type reported here to assess and monitor tampon safety in the future. While testing for one individual EDC with standard mass spectrometry approaches is useful, it will not provide a full account of potential estrogenic leaching. Encouragingly, the absence of detectable estrogenic bioactivity in some brands shows that a safer formulation is possible. While the relevance of these in vitro observations to human health is not yet clear, these findings highlight the need to better understand potential long-term exposure to EDCs associated with repeated use of menstrual products.

## Data Availability

The data supporting the findings of this study are available from the corresponding author upon reasonable request.

## Abbreviations

E2: estradiol
EDC: endocrine-disrupting chemical
ER: estrogen receptor
FBS: fetal bovine serum
LC-HRMS: liquid chromatography–high-resolution mass spectrometry
